# Integrated strength of osmotic potential and phosphorus to achieve grain yield of rice under water deficit by arbuscular mycorrhiza fungi

**DOI:** 10.1038/s41598-023-33304-x

**Published:** 2023-04-12

**Authors:** Suravoot Yooyongwech, Rujira Tisarum, Thapanee Samphumphuang, Muenduen Phisalaphong, Suriyan Cha-um

**Affiliations:** 1 School of Interdisciplinary Studies (Kanchanaburi Campus), Mahidol University, Kanchanaburi, 71150 Thailand; 2 National Center for Genetic Engineering and Biotechnology (BIOTEC), National Science and Technology Development Agency (NSTDA), Pathum Thani, 12120 Thailand; 3 Department of Chemical Engineering, Faculty of Engineering, Chulalongkorn University, Bangkok, 10330 Thailand

**Keywords:** Plant sciences, Arbuscular mycorrhiza

## Abstract

Arbuscular mycorrhizal ecosystem provides sustainability to plant integrity under drought situations. However, host plants that survive in drought frequently lose yield. The potential of *Funneliformis mosseae* (F), *Claroideoglomus etunicatum* (C), and *Acaulospora fovaeta* (A) was assessed to evaluate in indica rice cv. Leum Pua during booting stage under 21-day water withholding. The effects of three inoculation types; (i) F, (ii) F + C (FC), and (iii) F + C + A (FCA), on physiological, biochemical, and yield traits were investigated. The three types showed an induced total chlorophyll content in the host as compared to uninoculated plants. Total soluble sugars and free proline were less regulated by FC and FCA inoculated plants than by F inoculated plants under water deficit conditions. However, the FC and FCA inoculations increased phosphorus content, particularly in the shoots of water-stressed plants. In the three inoculations, the FCA dramatically improved plant osmotic potential adaptability under water deficit stress. Furthermore, even when exposed to the water deficit condition, panicle weight, grain number, and grain maturity were maintained in FCA inoculated plants. According to the findings, the increased osmotic potential and phosphorus content of the FCA-inoculated rice plant provide a protection sign against drought stress and will benefit food security in the future.

## Introduction

Drought stress is occurring frequently in the present scenario of global climate change^1,2^. In the past decade, drought stress has led to the reduction in crop yield by 70%^3^. Meteorological drought, agricultural drought, hydrological drought, and socio-economic drought are the various effect types of droughts that pose a threat to global food security^4,5^. In rice crop, poor grain production due to plant growth inhibition, grain sterility and yield loss upon the exposure of drought stress have been well established^6,7^. Proper irrigation, based on water requirement of individual crop, is one of the most common strategies used to sustain crop productivity^8–10^. Alternatively, addition of microorganism consortium to the soil has been reported as a sustainable way to improve soil physical and chemical properties, leading to more efficient water holding capacity and drought alleviation in the targeted area^11–14^.

Furthermore, climate change and food security are two of the world's most pressing issues^3^. The use of arbuscular mycorrhizal fungi (AMF) is one of the most profitable ways to improve plant development for long-term sustainability^15,16^. *Glomus mosseae*, also known as *Funneliformis mosseae*, is one of the most common AMF species used to improve chlorophyll content, photosynthetic efficiency, mineral nutrients, and shoot–root traits in host plants under stressful conditions, such as heavy metal^17^ and drought stress^18^. Besides, even under drought stress, a single inoculation of *Funneliformis mosseae* or *Funneliformis etunicatum* has additional benefits in terms of phosphorus regulation, iron (Fe) nourishment, and growth promotion in wheat^19^. In the case of an AMF consortium, a combination of several AMF species is frequently used to enhance its positive effects; for example, combining of *Funneliformis* plus with *Acaulospora* species significantly improves physiological and biochemical adaptabilities in *Sorghum bicolor* for drought^20^. Similarly, inoculation with a mixture of 25 AMF species from four genera, including *Funneliformis*, *Acaulospora*, *Gigaspora*, and *Scutellospora*, resulted in improved plant development^21^. AMF has been implicated in some mechanisms of a plant species' drought-tolerant abilities, including increased water and nutrient uptake, superior root architecture, higher root biomass, better osmotic adjustment, and antioxidant defense system enhancement^22–25^. However, a question of plant survival and reproductive regulation effectiveness remains in the AMF-host symbiosis under drought conditions^26,27^.

In AMF implementation in rice, lowland paddy fields flooded with water are usually unsuitable for AMF inoculation, and the diversity of AMF consortium suitable for these fields is determined by their anaerobic root zones^26,28,29^. In recent studies, non-flooded upland paddy cultivation was chosen as a model system to understand the AMF-plant interaction under water deficit stress^30–33^.

To investigate the regulation of physiological and biochemical attributes in rice (upland type) during the reproductive stage under water deficit, *Funneliformis mosseae* was chosen individually and in combination with *Claroideoglomus etunicatum* and *Acaulospora fovaeta*. According to the hypothesis, the AMF consortium with *Funneliformis mosseae* may be able to regulate host plant attributive mechanisms relating to grain production efficiency under water deficit stress.

## Results

### C*hanges in sugar and proline contents.*

**Figure 1:**
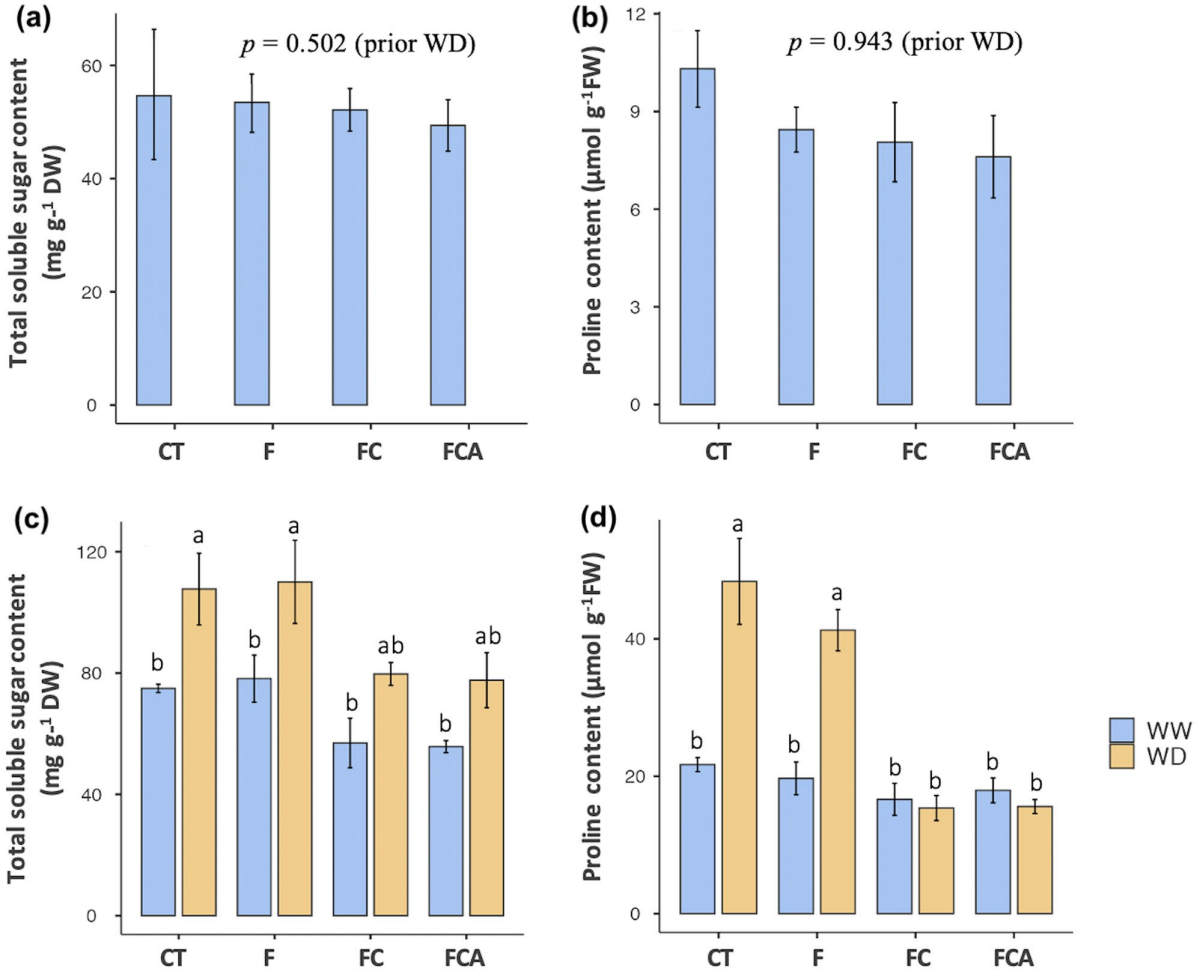
Total sugar soluble content and proline content of rice cv. Leum Pua inoculated with or without AMFs (*Funneliformis mosseae*; F, with *Claroideoglomus etunicatum*; C, and *Acaulospora fovaeta*; A, in F, FC, FCA, and uninoculated control, CT) under prior water deficit on day 0 (**a** and **b**) and day 21 of well-watered and water deficit conditions (**c** and **d**). Statistical data access with a minimum of n = 4 and mean ± standard deviation. Different letters along plots represent significant difference at *p* $=$ 0.05.

The AMF inoculations (F, FC, and FCA) were treated to the ‘Leum Pua’ rice under water deficit condition (WD). Under water well condition (WW), the total soluble sugar content of F inoculated and uninoculated plants was higher than that of FC and FCA-inoculated plants. Under WD stress, the total sugar content increased to 108, 110, 79.7, and 77.6 mg g^−1^ DW in the uninoculated, F, FC, and FCA plants, respectively (Fig. 1c). The soluble sugar content of FCA inoculated plants in the external reference control was found to be slightly lower, reflecting the previous stage of water deficit (Fig. 1a). Similarly, the free proline content in F inoculated and uninoculated plants under WD was recorded at 41.3 and 48.3 μmol g^−1^ FW, respectively, whereas the free proline content in FC and FCA plants was 15.4 and 15.6 mol g^−1^ FW, respectively (Fig. 1d). Furthermore, in all three AMF inoculations (F, FC, and FCA), the proline content in the external reference control decreased when compared to uninoculated plants (Fig. 1b).

### Enrichment of chlorophyll content, and growth

**Figure 2:**
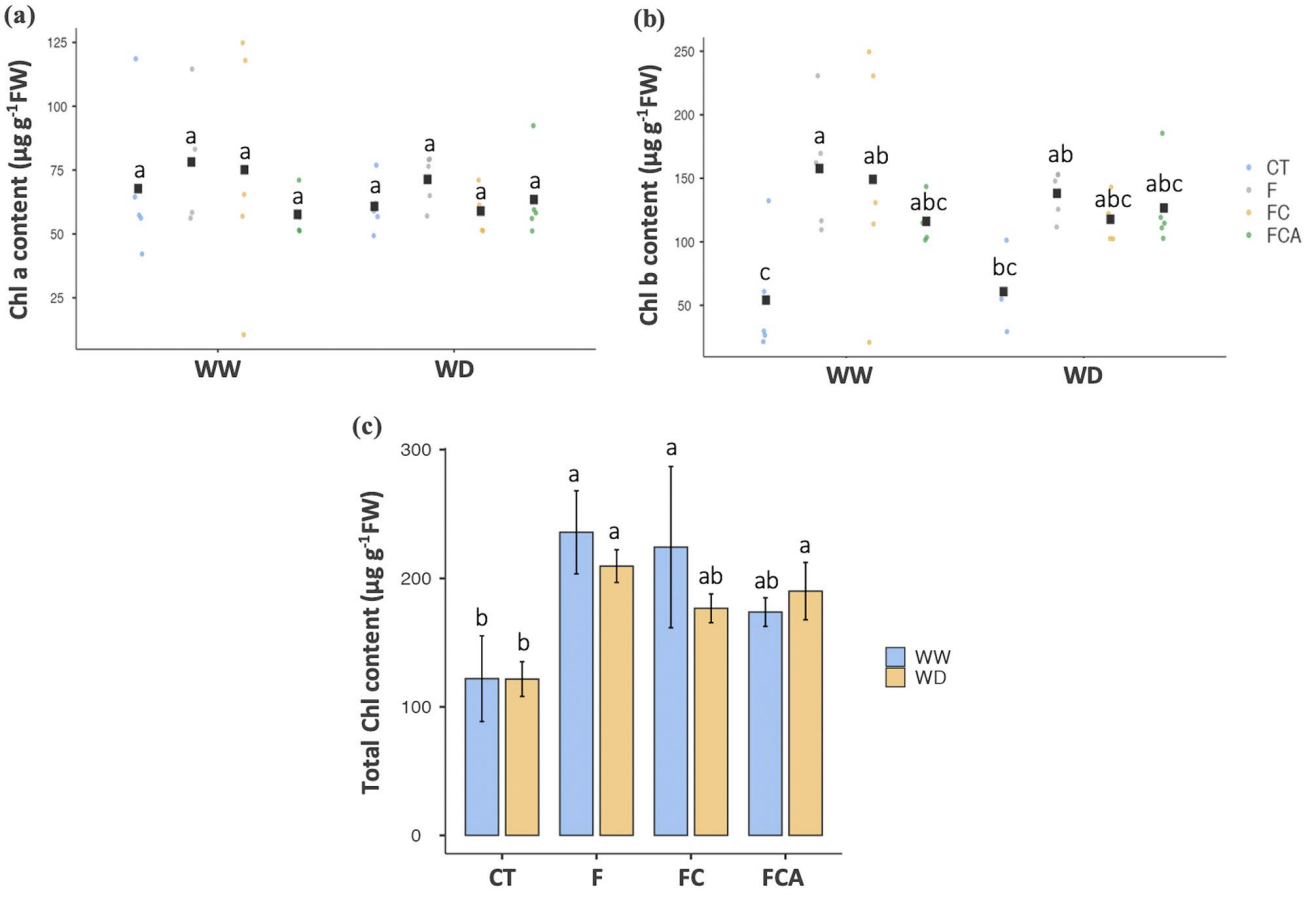
Chlorophyll, Chl *a* and *b* (**a** and **b**) and total chlorophyll content (**c**) in rice cv. Leum Pua that inoculated with or without AMFs (F, FC, FCA, and CT) on the 21st day of well-watered and water deficit conditions. Statistical data access with a minimum of n = 4 and mean ± standard deviation. Different letters along plots represent significant difference at *p* $=$ 0.05.

**Figure 3:**
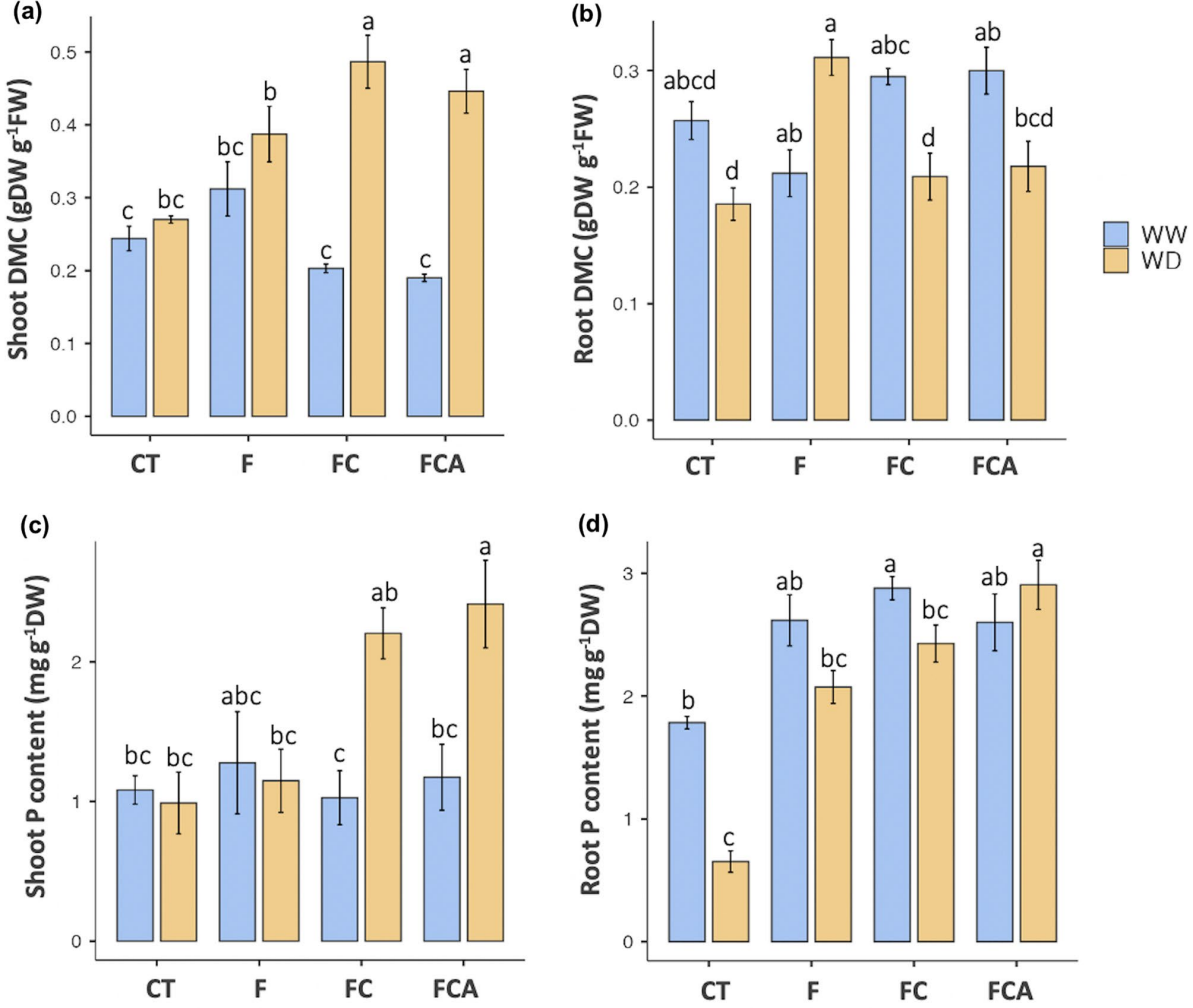
Dry matter content (DMC) of shoot and root (**a** and **b**) and phosphorus content in the shoot and root (**c** and **d**) in cv. Leum Pua inoculated with and without AMFs (F, FC, FCA, and CT) on the 21st day of well-watered and water deficit conditions. Statistical data access with a minimum of n = 4 and mean ± standard deviation. Different letters along plots represent significant difference at *p* $=$ 0.05.

In the case of photosynthetic pigments, the three AMF inoculations had a significant impact on chlorophyll *b*, which was present between chlorophyll *a* and *b*. (Fig. 2a,b). Under the experimental conditions, the effects of the three AMF treatments on total chlorophyll content in host plants were significantly improved (Fig. 2c). However, the total chlorophyll content of the F inoculated plant was higher than that of the FC and FCA-inoculated plants under the WD, with values of 209.53, 176.72, and 190.09 μg g^−1^ FW, respectively. In addition, plant height of the FCA inoculated plant was increased (9.53% over the control plant) under WD (Supplementary Table S1), while a reduction was demonstrated in the F and FC inoculated plants. Dry matter content (DMC) in the shoot of host plants under WD was enhanced relative to the WW condition by 29, 77, and 51% in F, FC and FCA inoculated plants, respectively. Whereas, the DMC in the root tissues under the WD was lower in the FC, FCA inoculated plants, but higher in the F inoculated plant (41% increment) (Fig. 3a,b).

### Variation of phosphorus content

When compared to the uninoculated plant, phosphorus content in the root was significantly improved in the F, FC, and FCA inoculated plants under two treatments, WW and WD. Whereas the shoot phosphorus content was higher in the FC and FCA inoculated plants under the WD, at 2.20 and 2.41 mg g^−1^ DW, respectively, compared to the F inoculated plant (1.15 mg g^−1^ DW). On the other hand, the phosphorus content in the shoots of the FC and FCA inoculated plants increased dramatically during the booting stage when exposed to the WD (Fig. 3c,d).

### Divergent osmotic potential

**Figure 4:**
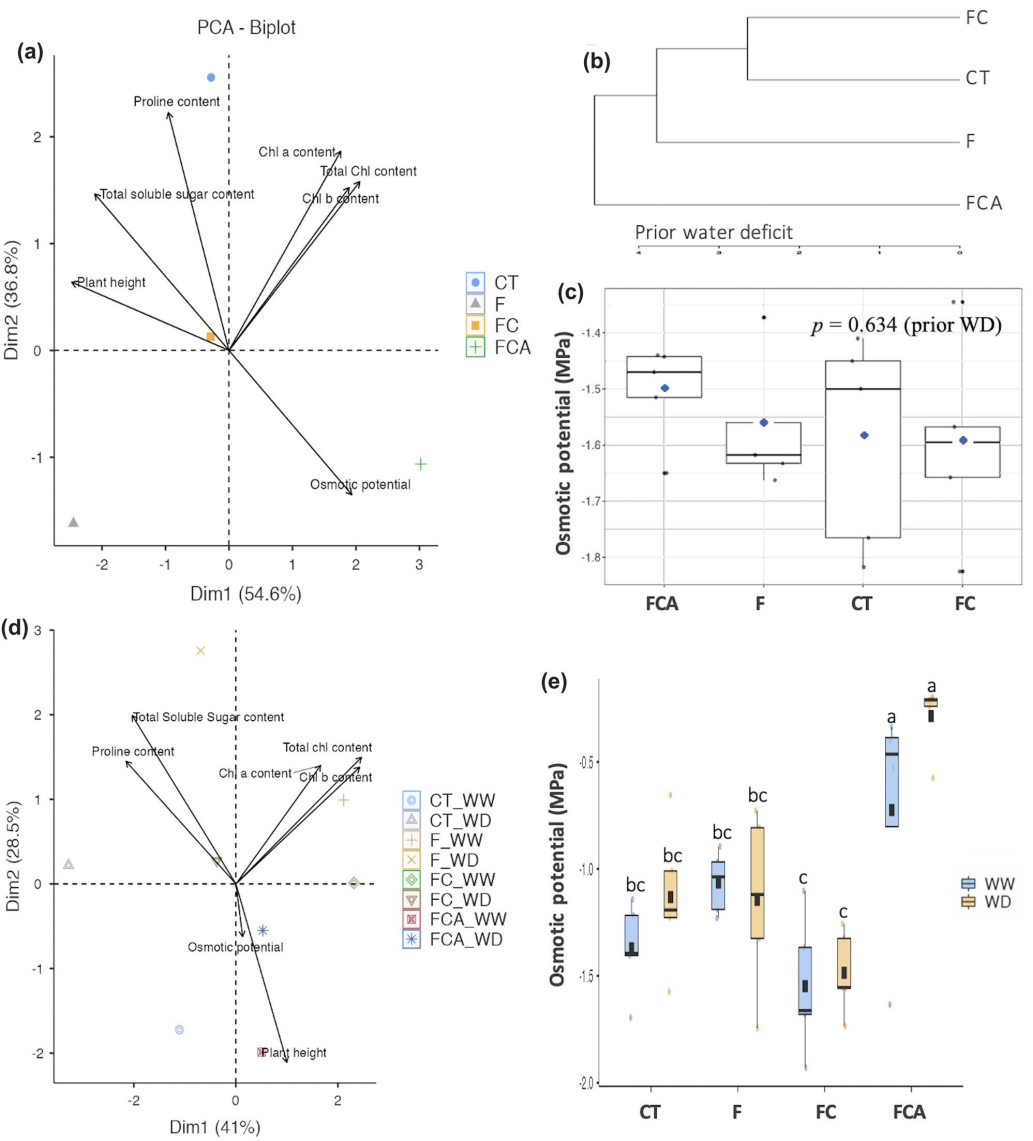
Principle component analysis, PCA, and group clustering (**a** and **b**) in rice cv. Leum Pua inoculated with or without AMFs (F, FC, FCA, and CT; the codes are included in Fig. 1) indicated by physiological, biochemical, and growth parameters, including plot of osmotic potential, data sorted by means, (**c**) under prior water limitation (0 day). The PCA (**d**) and the osmotic potential (**e**) represents on the 21st day of well-watered and water deficit conditions. Statistical data access with a minimum of n = 4 and mean ± standard deviation. Different letters along plots represent significant difference at *p* $=$ 0.05.

Principal component analysis (PCA) was performed in terms of physiology and growth, photosynthetic pigments, proline content, total soluble sugar content, osmotic potential, and plant height (Fig. 4a,d). In particular, an upregulation of osmotic potential was observed in the FCA inoculated plant prior to the WD (Fig. 4a,c). Consequently, the characteristics of the FCA inoculated plant were separated via hierarchical clustering, whereas the F and FC inoculated plants were approached more closely with the uninoculated plant (Fig. 4b). During the WD period, the osmotic potential of the FCA inoculated plant significantly increased and distinguished from the others (Fig. 4d,e). The FCA-inoculated plants had the highest osmotic potential among the three treatments, measuring − 0.73 and − 0.29 Mpa under the WW and WD conditions, respectively. Under WW and WD, the osmotic potential of the FC-inoculated plant was lower (− 1.55 and − 1.49 MPa) than that of the F inoculated plant (− 1.06 and − 1.15 MPa) (Fig. 4e).

### Reproductive yields and harvesting quality

**Figure 5:**
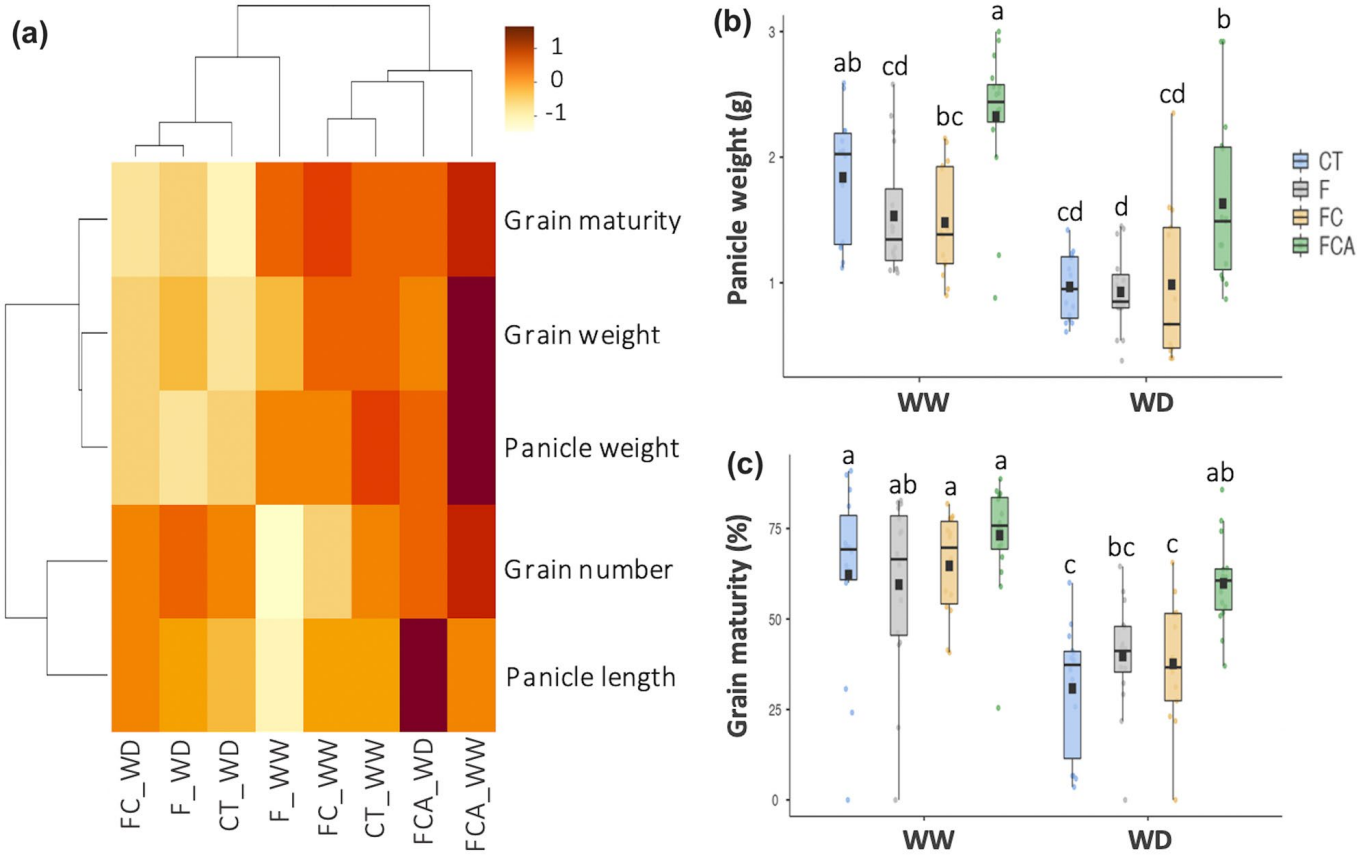
Heat map correlation of panicle and grain production/quality (**a**), and plot of panicle weight and grain maturity per panicle (**b** and **c**) of rice cv. Leum Pua inoculated with or without AMFs (F, FC, FCA, and CT) on the 21st day of well-watered and water deficit conditions. Statistical data access with n = 15 and mean ± standard deviation. Different letters along plots represent significant difference at *p* $=$ 0.05.

Reproductive parameters, such as panicle weight, panicle length, grain weight, grain number per panicle, and percentage of grain maturity per panicle were maintained in the FCA treatment upon under the WD condition (Fig. 5a). In contrast, the F and FC inoculated plants under the WD had a non-dominant panicle weight and percentage of grain maturity compared to the FCA inoculated plant under the WD (Fig. 5a–c, Supplementary Fig. S2). Furthermore, an order of these eight plant treatments within the reproductive parameters, from FC_WD to FCA_WW, in Fig. 5a appeared to correspond to the groups of score plot of these plant treatments in Fig. 4d such as group of CT_WW, FCA_WW, and FCA_WD etc.

### Different of the physiological, biochemical, and reproductive responses

**Figure 6:**
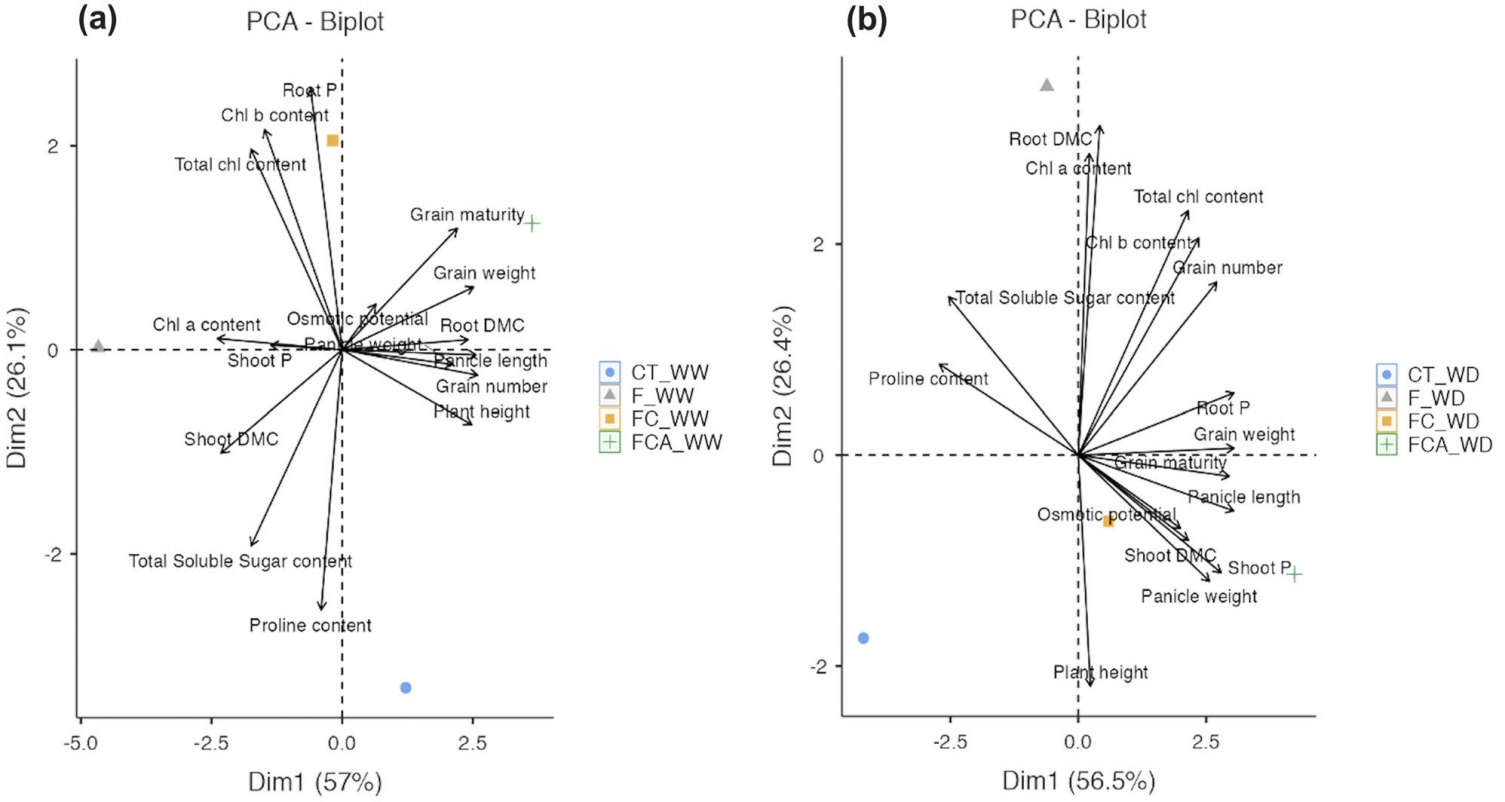
Principle component analysis depicting score and loading plots of the experimental physiological and biochemical parameters, growth, panicle-grain production/quality, and dry matter content (DMC) in rice cv. Leum Pua inoculated with or without AMFs (F, FC, FCA, and CT) and on the 21st day of well-watered (WW, **a**) and water deficit (WD, **b**) conditions.

**Figure 7:**
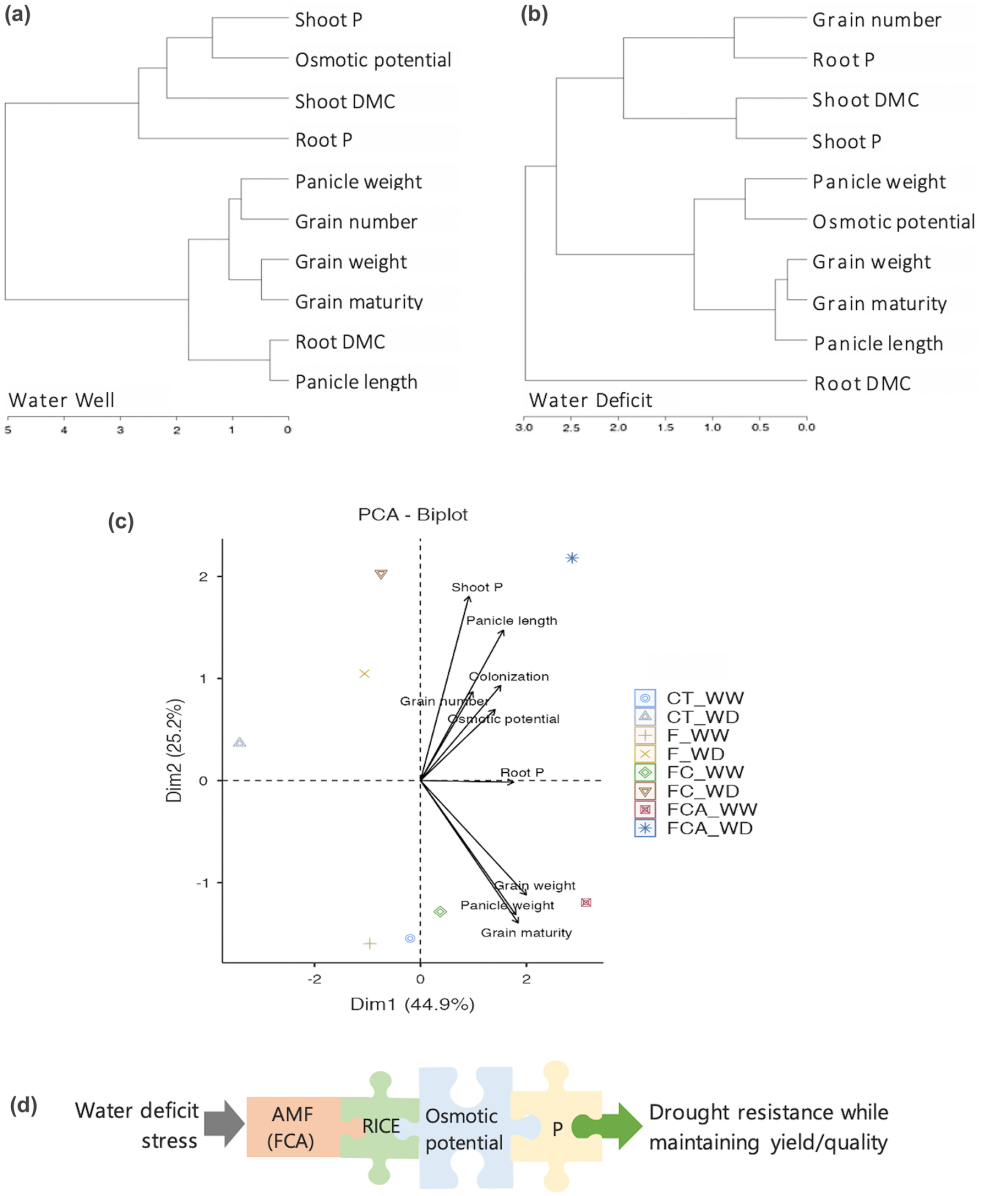
Clustering of separated well-watered (**a**) and water deficit (**b**) conditions in relationship of osmotic potential and phosphorus content into the reproductive parts (panicle – grain production, quality) and vegetative (dry matter content, DMC, of shoot and root) parts in rice cv. Leum Pua inoculated with or without AMFs (F, FC, FCA, and CT). PCA summary (**c**) in effect of the AMF colonization on osmotic potential, phosphorus (P) and reproductive yields. Systematic diagram jigsaws (**d**) depicting the effect of osmotic potential and phosphorus regulation by the AMF (FCA type) on rice yield maintenance under water deficit conditions.

Summarized PCAs reduced the dimensions of parameters such as osmotic potential, phosphorus content, chlorophyll, free proline, growth performance, yield traits such as plant height, shoot and root DMC, and the reproductive traits such as panicle length and panicle weight, percentage of grain maturity, and grain number and weight. Strength of these loading vectors suggests that reproductive traits are more closely associated in the FCA inoculated plant in the two water regimes than in the FC and F inoculated plants (Fig. 6a,b). By the way, the F inoculated plant performed well through the yield trait in the vegetative part of root DMC under the WD, when compared to the FC and FCA inoculated plants (Fig. 6b). According to the summarized PCA, the relationship between the phosphorus content and the osmotic potential within the vegetative and reproductive yields was depicted in Fig. 7. In the three AMF treatments, the phosphorus content and osmotic potential were found to not to be directly related to the reproductive yields under the WW. At the site of the WD, the osmotic potential and phosphorus content, especially in root, were closely related to panicle weight and grain number, respectively (Fig. 7a,b).

While emphasizing the influence of the AMF colonization on the osmotic potential, phosphorus content, and reproductive yields. The findings revealed a high relationship between the root phosphorus content and the colonization in the WW and WD conditions (Pearson coefficient; R > 0.8, Supplementary Table S2). Furthermore, when compared to the three inoculations in the WW and WD, the FCA inoculation showed a trend to towards the colonization influence rather than the others (Fig. 7c). It may involve a slightly high level in the percentage of the FCA colonization under the WW and WD (Supplementary Fig. S1). However, the three inoculated plants showed the different regulation, especially in the plant physiology and yields.

## Discussion

Both single and consorted inoculations of AMF have been used to promote plant survival under drought stress^27,34^. In the present study, the water deficit stressed plants of rice cv. Leum Pua at booting stage were treated with different AMF inoculations, i.e., F (*Funneliformis mosseae*), FC (F and *Claroideoglomus etunicatum*), and FCA (F, C and *Acaulospora foveate*). Total soluble sugar content and free proline content in the F-inoculated plant under the WD were highly accumulated, among the three inoculated plants. In the case of the F inoculated plant, the adjustments of soluble sugars and proline should result in the stability of the NADP + /NADPH ratio, which acts as osmoprotectants to avoid stress in the host plant under drought stress^21,35,36^. Furthermore, the free proline subsequence stimulates energy for root biomass accumulation, which improves water accessibility in soil^37^. Only the F inoculated plant showed the increase in the root biomass when compared to the other inoculated plants. Cheng et al.^18^ previously reported that the root traits were enhanced by *Funneliformis mosseae* symbiosis in trifoliate orange under drought condition. This indicates that F inoculation could help promote root development in rice crops via catalytic function and energy regulation.

Responses of the FC and FCA inoculated plants under the WD seem to be in contrast with the F inoculated plant, due to the lower accumulation of total sugar soluble content and free proline. The total sugar soluble content of FC and FCA inoculated plants in the WD was more upregulated than in the WW condition. Lower variation in the free proline content was also found in mycorrhizal symbiosis in macadamia, and thus, total soluble sugar was suggested as the major osmolyte^21^. Furthermore, Wu et al.^38^ announced that the osmolytes for water balance in plant cells may also originate from total non-structural carbohydrates and ions, *i.e.*, K^+^, Ca^2+^ and Mg^2+^, such as mycorrhizal inoculated citrus. In this case, the lower free proline content under drought was related to the proline biosynthesis and turnover reflected in buffer cellular redox status, resulting in maintenance of plant growth during water shortage^39^. Besides, the low carbohydrate accumulation in plant ‘source’ under drought (post-anthesis stage) could result in the growth of ‘sink’ in their shoots, such as panicle dry weight in Sorghum^40^. Between the free proline and carbohydrate content, here, the soluble sugar content might be required to generate a cellular homeostasis balance for the FC and FCA inoculated plants under the WD, involving drought susceptibility.

From the results of this study, the rice plants inoculated with the F only and those with two AMF consortiums are confirmed to have better chlorophyll pigments under both of the WW and WD. In agreement, the maize-*Funneliformis mosseae* symbiosis has been reported to lead to an increased chlorophyll content under irrigation^41^^.^ Furthermore, *F. mosseae* has been proposed as a preferred partner in physiological traits in C3 plants, including photosynthetic pigments^42^. It is suggested that *F. mosseae* has the potential to be a major inoculation for chlorophyll pigment improvement in plants.

In general studies of AMF colonization and phosphate induction^15^, the three AMF symbiotic relationships in the WW and WD were confirmed here, and their priorities were correlated to phosphorus content in the plant host, especially in the rice root (Supplementary Table S2). However, the phosphorus content in the shoot and root of the FC and FCA inoculated plants under the WD was higher than in the F inoculated plant. In particularly, the phosphorus content in the shoot was remarkably enriched by the combinations of FC and FCA inoculation. The results are related to the report by Kobae^43^ who reported that the mosaic of diverse mycorrhizas led to an increased ability of phosphate-uptake performance, and it is possible that the common mycorrhizal networks (CMNs) might share cellular components such as nuclease and other organelles in the coenocytic mycelia. There has been a tangible association in mycorrhizal communities between mycorrhizal functional diversity and plant nutrient stoichiometry, including phosphorus^44,45^. As the results of the improvement of phosphorus content, the FC and FCA are thought to be more related to rice nutrient availability under the WD. Phosphorus concentration helps in regulation of shoot biomass, which is important for the production of high fertile spikes and better grain yield in winter wheat^46^^.^ Thus, the increased plant growth and yield traits are strongly correlated with the increase in phosphorus uptake in AMF inoculated plants^47,48^. Then, the phosphorus content in both the FC and FCA inoculated plants is anticipated to be associated with shoot biomass, relating vegetative dry matter content and reproductive yield.

Despite the phosphorus replenishment, the FC inoculated plant did not improve its grain yield traits under the WD when compared to the FCA inoculated plant. This may involve in the difference of the osmotic potential in the FC and FCA plants as shown in Fig. 4. The upregulation of osmotic potential in the FCA inoculated plant should due to FCA combination, producing remarkable reproductive traits, especially those related to panicle and grain yield under the WD and WW conditions. Previous research found that osmotic potential and phosphorus content in WD were closely related to reproductive traits in *Spartina alterniflora* (when compared to WW)^49^. Sawwan et al.^50^ suggested that an upregulation of osmotic potential was very efficient under enhanced phosphorus content, as indicated in cell sap of an African violet. Phosphorus also induces a cell hydraulic state in the leaf tissues^51^. Based on the findings of the previous studies and the present study, it can be suggested that the FCA colonization should stimulate phosphorus accumulation, particularly an increase in osmotic potential during drought, which may facilitate solute allocation from root to shoot and panicle, potentially leading to drought resistance with reproductive yield, as shown in Fig. 7 in the FCA inoculated plant under the WD. The balance of the osmotic potential and phosphorus content are supposed to be important regulation for maintaining plant growth and grain development under WD stress in rice species. There is a high possibility that an effective AMF inoculation strategy might secure a reproductive rice yield during a drought situation.

## Conclusions

The potential of the consortium of AMF *Funneliformis mosseae* in the upland rice, Leum Pua cultivar, under water deficit was demonstrated. Total chlorophyll content in the flag leaf of rice inoculated with F, FC, and FCA was promoted under WD. However, the responses of total soluble sugar and free proline were upregulated in the F-inoculated plant and downregulated in FC and FCA-inoculated plants, leading to drought regulations. Both FC and FCA inoculations could be used for a remarkable increase in the phosphorus level, especially in the leaf tissues of host plants under WD stress. In addition, the combination of FCA resulted in osmotic potential adaptability in the host plant under the WD condition. It is concluded that the increased osmotic potential and phosphorus content of the FCA inoculated rice plants provide protection against water deficit stress. Therefore, further development of drought-resistant crops should focus on regulatory signaling mechanisms involved in osmotic potential and phosphorus adjustment.

## Materials and methods

### Preparation of plant materials and AMF inoculation

Rice (*Oryza sativa* subsp. indica; cv. Leum Pua; upland black sticky rice) seeds procured through collection by the Innovative Plant Genetic and Physiology Research, National Center for Genetic Engineering and Biotechnology (BIOTEC), Thailand, were selected as the initial plant material in the present study. The common and predominant cultivar of black sticky rice used in the study is identified as glutinous rice^52^ and enriched with anthocyanins^53^. Rice seeds were sown on the soil substrate for a month. Rice seedlings were directly transferred into 10 × 12.5 × 15 cm (width × length × height) of plastic bags filled with autoclaved garden soil (EC = 2.7 dS m^‒1^; pH = 5.7; total organic carbon = 12.3%; available N = 0.3 mg kg^‒1^; available P = 578 mg kg^‒1^; available K = 3073 mg kg^‒1^; available Ca = 7020 mg kg^‒1^; available Mg = 1034 mg kg^‒1^). According to arbuscular mycorrhiza fungi, the mycorrhiza powder of *Funneliformis mosseae* (F), *Claroideoglomus etunicatum* (C) and *Acaulospora fovaeta* (A) were obtained from Maejo University. All the three AMF have been primarily tested as single purified strains on a monocot species and the F showed the maximum benefit to their host^41^. On this basis individual F and consortium of FC, and FCA were used as treatments in the present study. For AMF inoculation, average 200 spores of each AMF were used. These AMFs and an organic fertilizer (Bua® chicken yard manner, Charoen Pokphand Group)^54^ were provided at the time of seedling transplantation. Then, the rice plants, at V_4_ stage; formation of four leaf on main stem, in the plastic bags were settled in 4 × 2 factorials in completely randomized design (CRD) of four inoculated treatments (control, and the three of AMFs) and two conditions of water regime (water well, WW and water deficit, WD). The rice was cultivated under the greenhouse conditions until its transition to the booting stage. The temperature in the greenhouse was set at 26 ± 2 °C (nighttime)/32 ± 2 °C (daytime) and the relative humidity at 80 ± 5%. After the booting stage, the uniform rice for 20 plants were selected at day 0 (day of starting in water withholding). The 40 bags (ten bags per each AMF treatment) were divided into a group of WW which continue watering, and a group of WD. The group of WD was withheld the water until 21 days. At the 21st day, the percentage of soil moisture content (by drying method^21^) was reported to 55.76 ± 3.12% for the WW, and 14.84 ± 2.14% for the WD. The plant samples of the two water-regimes were collected. Both sample collections of the 0 day and 21 days were used for physiological, biochemical, and morphological growth trait measurements. While, other 40 bags in the WW, and WD (re-watering after 21 days) conditions remained for yield traits at the harvesting period.

### Determination of total soluble sugar and free proline content

Total soluble sugar content was determined from the flag leaf of rice. Fifty milligrams of flag leaf samples were ground with liquid nitrogen. One mL of nano-pure water was added to the sample for extraction in a 1.5 mL plastic tube that modified from Karkacier et. al.^55^. After centrifugation at 10,000 × *g* for 15 min, the extracted solution was collected and filtrated through a 0.45 μm membrane filter (VertiPure™, Vertical®). Then, the 20 μL of filtrated supernatant was analyzed by a High-Performance Liquid Chromatography (HPLC) system (Waters^TM^410, Massachusetts, US) equipped with a MetaCarb 87C column, using a differential refractometer detector. A mobile phase was deionized water, and the flow rate was set at 0.5 mL min^−1^. The standard curves of sucrose, glucose, and fructose (Fluka, US) were used to calculate total soluble sugar content^55^.

The analysis of free proline content was conducted with the flag leaf samples. The freeze-dried flag leaf tissues were ground to the powder using liquid nitrogen, and 50 mg of sample were mixed into 1 mL aqueous sulfosalicylic acid (3%, *w/v*). The supernatant was separated and added with the same volume of glacial acetic acid and ninhydrin reagent, then heated at 95 °C in a water bath for 1 h and kept on an ice box to terminate the reaction for 15 min. The solution was then mixed with 2 mL of toluene to precipitate the free proline, and the chromophore absorbance at 520 nm was measured on a UV–Vis spectrophotometer (HACH DR/4000; Model 48,000, HACH Company, Loveland, Colorado, US). The content of free proline was estimated against the L-proline standard curve between 0 and 500 μM^56^.

### Elucidation of chlorophyll content

Analysis of chlorophyll content was performed as per the protocol modified from Shabala et al.^57^ and Lichtenthaler et al.^58^. The flag leaf samples were collected and chopped into small pieces. After that, 100 mg of sample were transferred to a 25 mL glass vial (Opticlear®; KIMBLE, Vineland, NJ, USA), 10 mL of 95.5% acetone was added, and the mixture was homogenized with a homogenizer (T25 basic Ultra-Turrax®; IKA, Kuala Lumpur, Malaysia). The solution was kept in the sealed glass vial with a plastic cap and incubated at 4 °C in the refrigerator for 48 h. The extracted solution was read by a UV–VIS spectrophotometer (DR/4000; Model 48000Hatch, Loveland, CO, USA) at 662 and 645 nm, and then chlorophyll *a*, *b* and total chlorophyll content were calculated.

### Analysis of phosphorus content

The plants were separated into shoots and roots, and oven-dried at 80 °C for 3 days. The 0.5 g of the powdered samples was digested by a nitric acid solution using microwave digestion. The digested solutions were subjected to molybdenum blue reaction and their absorbance was read at 420 nm using spectrophotometer [DR/4000; Model 48,000, (HACH.), USA]^59^.

### Osmotic potential determination

Osmotic potential was investigated in according to Lanfermeijer et al.^60^ method. Hundred milligrams of fresh flag leaf samples were manually crushed using glass rod in 1.5 mL plastic tube, and the 20 μL of the extracted solution was directly dropped on a filter paper and incubated osmometer chamber (5520 Vapro®, Wescor, Utah, USA). Finally, the millimolar per kg of osmotic potential data was converted into osmotic potential in MPa in according to Fu et al.^61^.

### AMF colonization and growth measurement

According to Brundrett et al.^62^, root samples of the rice were cleaned with tap water, followed by distilled water. In 60 percent ethanol, one centimeter of the root sample was reserved. The root was then washed three times with distilled water before being immersed in 10% KOH at 95 °C for 30 min. After that, the cleaned root was processed with 0.05% (w/v) Trypan blue for 15 min. The AMF colonization was observed under a light microscope (Zeiss, Germany) and calculated in percentage (Supplementary Fig. S1).

Plant height and dry mass per fresh mass in the rice shoot and root were measured following the IRRI protocol^63^. At the harvesting stage, panicle length, panicle weight, total grain number per panicle, grain weight, and percentage of grain maturity by filled grains per panicle were evaluated.

### Data analytics and statistical analysis

According to the information and Table 1, the data were statistically analyzed using Jamovi (v. 2.2) (https://www.jamovi.org). The physiological, biochemical, and morphological data in the present study were analyzed at least four technical replications, a replication per individual plant sample. The plant sampling was varied among each rice plant in those groups of treatments. In reproductive traits, fifteen panicles, per treatment, were used for yield traits. External references included the total soluble sugar content, proline content, and osmotic potential at the beginning (zero day; prior day of water deficit). The external reference was subjected to principal component analysis (PCA) and hierarchical clustering. Induvial box plot was shown for osmotic potential and yield traits with mean (dot), median line, at 25–75% of interquartile range with standard deviation calculation. A heat map dendrogram was created for rice production under WW and WD conditions. PCA was used to summarize the effect of water stress on physiological and biochemical factors, including parts of yield traits and colonization percentage. The dendrograms displayed phosphorus content and osmotic potential data related to rice production. Furthermore, Tukey's honestly significant difference was used in SPSS v.18 to identify a post-hoc comparison test with analysis of variance.

**Table 1:** Analyses of variance depicting the effect of inoculated treatment (T), two watering conditions (Con), and their interaction on the physiological and biochemical parameters in the 21-day water limitation and the production in harvesting state. The numbers indicate significant difference, and ns represent non-significant difference.

Parameter test	T	Con	T $\times$ Con
Total soluble sugar content	0.003	< 0.001	ns
Proline content	< 0.001	< 0.001	< 0.001
Chlorophyll a content	ns	ns	ns
Chlorophyll b content	< 0 .001	ns	ns
Total chlorophyll content	0.013	ns	ns
Shoot dry matter content	0.020	< 0.001	< 0.001
Root dry matter content	ns	0.012	< 0.001
Shoot phosphorus	0.018	0.004	0.009
Root phosphorus	< 0.001	< 0.001	0 .001
Osmotic potential	< 0.001	ns	ns
Panicle weight	< 0.001	< 0.001	ns
Grain maturity	< 0.001	< 0.001	ns

### Complies with international, national and/or institutional guidelines

The experimental research and field studies on plants, including materials, reported here comply with all relevant institutional, national, and international guidelines and legislation. 

## Supplementary Information

Supplementary Information.

## Data Availability

The data underlining this article are included in the manuscript, and the raw data will be made available to the corresponding author upon reasonable request.
