# Supplementary material for: Integrated strength of osmotic potential and phosphorus to achieve grain yield of rice under water deficit by arbuscular mycorrhiza fungi
Source: Sci Rep. 2023 Apr 12;13:5999. doi: 10.1038/s41598-023-33304-x (PMC10097676; doi:10.1038/s41598-023-33304-x)
Supplement: Supplementary file 1 — Supplementary Information. [file 41598_2023_33304_MOESM1_ESM.pdf]

Supplementary Information

In

Integrated strength of osmotic potential and phosphorus to achieve grain yield of rice under water deficit by arbuscular mycorrhiza fungi

By Suravoot Yooyongwech[1]*, Rujira Tisarum[2], Thapanee Samphumphuang[2], Muenduen Phisalaphong[3] and Suriyan Cha-um[2]

[1]School of Interdisciplinary Studies (Kanchanaburi Campus), Mahidol University, Kanchanaburi 71150, Thailand. [2]National Center for Genetic Engineering and Biotechnology (BIOTEC), National Science and Technology Development Agency (NSTDA), Pathum Thani 12120, Thailand. [3]Department of Chemical Engineering, Faculty of Engineering, Chulalongkorn University, Bangkok 10330, Thailand. *e-mail: suravoot@gmail.com

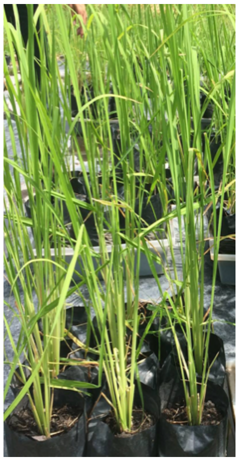

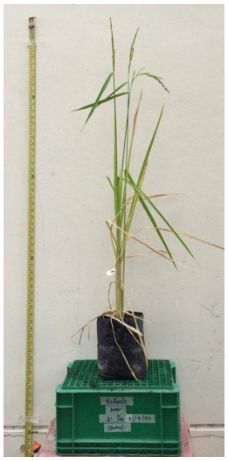

Treatment	Plant height (cm)	
	**WW**	**WD**
CT	77.30 ± 1.76a	49.3 ± 1.60c
F	66.20 ± 2.33ab	23.40 ±1.43d
FC	74.20 ± 1.32a	23.6 ± 2.25d
FCA	78.60 ± 3.44*a*	54.0 ± 6.63bc
**ANOVA**	0.01**	

Table S1. Table data of plant height in rice cv. Leum Pua that inoculated with or without AMFs (*Funneliformis mosseae*; F, with *Claroideoglomus etunicatum*; C, and *Acaulospora fovaeta*; A, in F, FC, FCA, and uninoculated control, CT) on the 21st day of well-watered and water deficit conditions. Pot plant in the experiment (above).

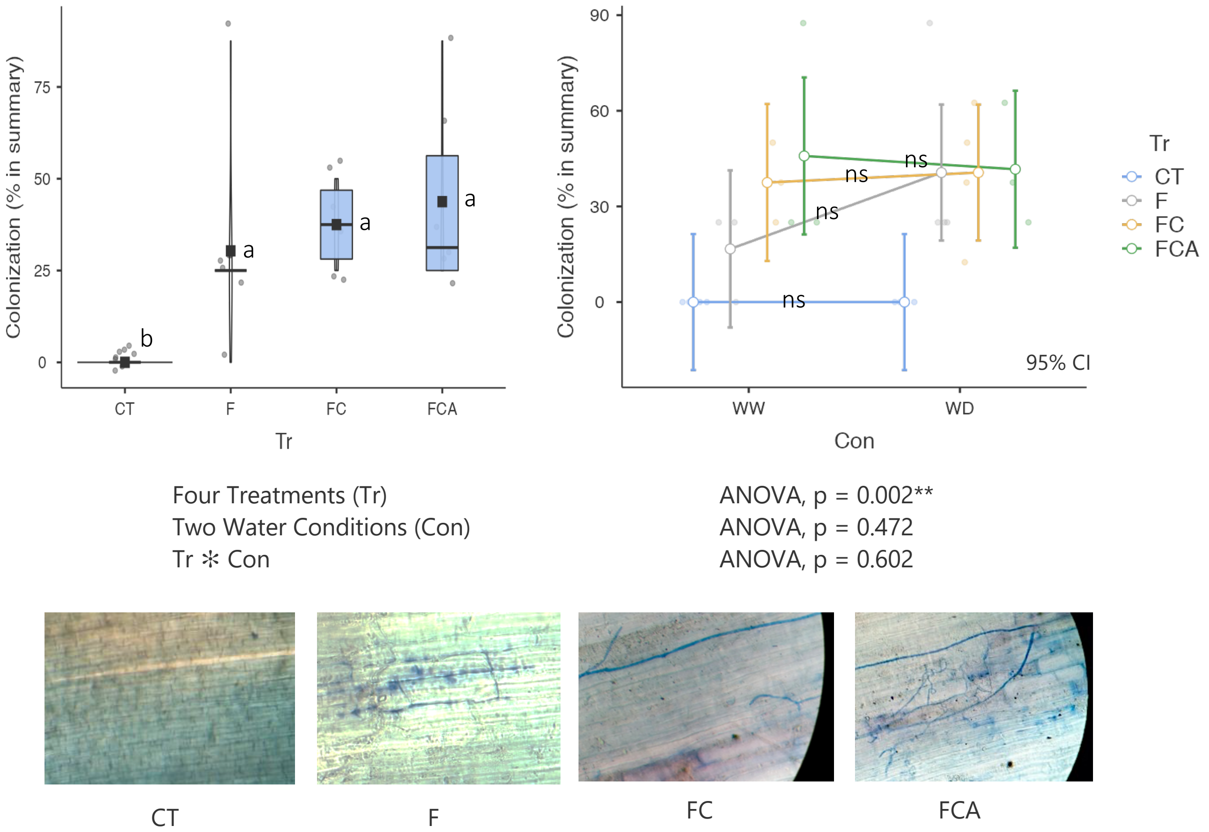

Figure S1. Percentage of root colonization in rice cv. Leum Pua that inoculated with or without AMFs (*Funneliformis mosseae*; F, with *Claroideoglomus etunicatum*; C, and *Acaulospora fovaeta*; A, in F, FC, FCA, and uninoculated control, CT). The percentage of colonization in each AMF treatment in the water (WW plus WD) conditions is summarized in box plots with means and median lines. The colonization when comparing the WW and WD in an estimate marginal mean with 95% confidence intervals, at least n = 3. The image of the root section with and without the AMFs.

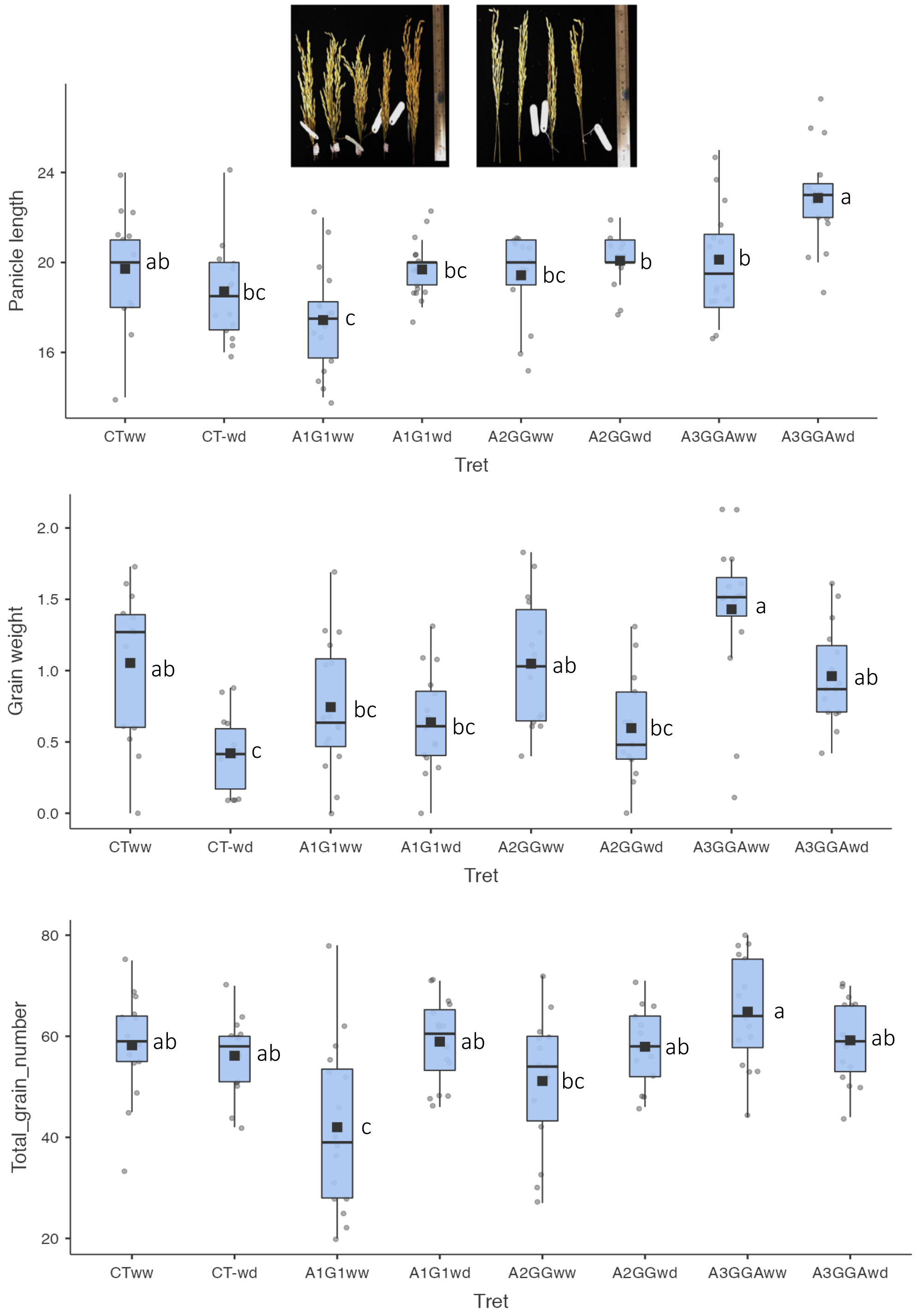

Figure S2. Panicle length, grain weight, and total grain number per panicle in rice cv. Leum Pua was inoculated with or without AMFs (*Funneliformis mosseae*; F, with *Claroideoglomus etunicatum*; C, and *Acaulospora fovaeta*; A, in F (equal A1G1), FC (equal A2GG), FCA (equal A3GG), and uninoculated control, CT) on the 21st day of well-watered (WW) and water deficit (WD) conditions.

**Pearson coefficient correlation**		Colonization		
		SUM (WW+WD)	WW	WD
Colonization	Pearson's r	—	—	—
	p-value	—	—	—
Osmotic potential	Pearson's r	0.281	0.382	0.175
	p-value	0.500	0.618	0.825
Shoot P	Pearson's r	0.462	-0.061	0.657
	p-value	0.249	0.939	0.343
Root P	Pearson's r	**0.741***	**0.805**	**0.943**
	p-value	0.036	0.195	0.057
Panicle length	Pearson's r	0.510	0.317	0.631
	p-value	0.196	0.683	0.369
Grain number	Pearson's r	0.336	0.304	0.924
	p-value	0.416	0.696	0.076
Grain maturity	Pearson's r	0.241	0.773	0.619
	p-value	0.565	0.227	0.381
Grain weight	Pearson's r	0.355	0.599	0.708
	p-value	0.389	0.401	0.292
Panicle weight	Pearson's r	0.113	0.336	0.340
	p-value	0.790	0.664	0.660

Table S2. Pearson coefficient correlation of colonization data within osmotic potential, phosphorus content (shoot and root), and those five reproductive yield traits in Leum Pua rice host in summary (WW plus WD), WW, and WD conditions (* $p < .05$).
